# Microbiota-mediated protection against antibiotic-resistant pathogens

**DOI:** 10.1038/s41435-021-00129-5

**Published:** 2021-05-04

**Authors:** Rekha B. Panwar, Richard P. Sequeira, Thomas B. Clarke

**Affiliations:** MRC Centre for Molecular Bacteriology and Infection, Department of Infectious Disease, Imperial College London, London, UK

**Keywords:** Infection, Innate immune cells, Mucosal immunology

## Abstract

Colonization by the microbiota provides one of our most effective barriers against infection by pathogenic microbes. The microbiota protects against infection by priming immune defenses, by metabolic exclusion of pathogens from their preferred niches, and through direct antimicrobial antagonism. Disruption of the microbiota, especially by antibiotics, is a major risk factor for bacterial pathogen colonization. Restoration of the microbiota through microbiota transplantation has been shown to be an effective way to reduce pathogen burden in the intestine but comes with a number of drawbacks, including the possibility of transferring other pathogens into the host, lack of standardization, and potential disruption to host metabolism. More refined methods to exploit the power of the microbiota would allow us to utilize its protective power without the drawbacks of fecal microbiota transplantation. To achieve this requires detailed understanding of which members of the microbiota protect against specific pathogens and the mechanistic basis for their effects. In this review, we will discuss the clinical and experimental evidence that has begun to reveal which members of the microbiota protect against some of the most troublesome antibiotic-resistant pathogens: *Klebsiella pneumoniae*, vancomycin-resistant enterococci, and *Clostridioides difficile*.

## Introduction—microbiota development and importance

In humans, and other mammals, all surfaces exposed to the environment are home to myriad archaea, bacteria, viruses, and eukaryotic microbes [1, 2]. These colonizing microbes are referred to as the microbiota. For their hosts they provide a variety of benefits, including the provision of nutrients, protection against infections, and maturation of the immune system [2, 3]. In exchange these organisms are provided with a nutrient-rich habitat [1]. The principal system we have for interacting with the microbiota is the immune system [4–7]. The central role of these microbes in programming our immune system, and host health more broadly, has been demonstrated in both human and animal studies, where microbiota disruption or depletion has been linked to a range of diseases and immune dysfunctions, including autoimmunities [8–10] and increased susceptibility to infection by all classes of pathogens [4, 5, 11–17]. Better understanding how host and microbiota interact may therefore allow us to minimize disruption to the microbiota to prevent disease, and also harness the power of the microbiota to treat disease.

The microbiota develops in an ordered manner with transitions that mirror many of the major steps in host development [18–21]. Prior to birth, host mucosal and skin epithelia are not thought to be colonized by live microbial communities, then, immediately after birth, the process of microbiota colonization begins [22, 23]. In newborns all skin and mucosal epithelia have a similar microbiota composition and it is thought that this initial seeding microbiota is derived from the mothers’ vaginal and intestinal microbiota [2, 23–25]. By contrast in babies born by caesarian section the microbiota of the newborn does not reflect the mothers’ vaginal microbiota but more closely resembles her skin microbiota for the first few months of life [18, 26, 27]. This highlights how swift the acquisition of the microbiota is during the birthing process and how amenable the mucosa of newborns is to microbial colonization. Over the next 2–3 years the microbiota of each different mucosal site develops and differentiates such that each mucosal site has its own unique microbiota [19]. The balance of factors that shape the microbiota is thought to reflect the environmental, nutritional, microbial, and immunological challenges at each site. In the intestine, for example, the process of microbiota development is strongly influenced by host diet, with the lactobacilli that are part of the seeding inoculum continuing to dominate until weaning where the transition to solid foods corresponds to the development of more anaerobic bacterial communities from the Bacteroidia and Clostridia taxa [1, 17, 28, 29]. In the upper respiratory tract, the process of differentiation can occur quickly, beginning after only a week of life but the factors driving this are less clear [30, 31]. In parallel to microbiota development the development of immune system occurs. Many aspects of the production and functional maturation of the immune cells are shaped by the acquisition of the microbiota in tissues throughout the body [4, 5, 32–34]. The parallel development of the microbiota and immune system suggests a reciprocal relationship whereby not only does the microbiota drive immune development but also that the immune system can shape and control the microbiota [35]. The orchestrated patterns of immune and microbiota development that occurs in mammals result in the establishment of a highly effective barrier against infectious disease in adulthood [3, 5, 36]. Host resistance to infection is therefore established by the microbiota either through its direct antimicrobial activity or indirectly through its regulation of the immune system, particularly the innate immune system.

## The microbiota as a regulator of innate immune development and function

The innate immune system is the first arm of the immune system to respond to microbial infection [37]. Historically, the production and functional maturation of the cells that constitute this branch of the immune system have been thought of as controlled by host signals alone [37–39]. This view has been increasingly revised as our understanding of the role played by the microbiota in mammalian physiology increases [6, 40]. It is now clear that many aspects of innate immune cells have their function modified by the microbiota. The epithelium is the hosts’ first tissue to interact with the microbiota, it must therefore act as a barrier to prevent invasion of the microbiota into deeper, more sensitive host tissues. Its role is not simply limited to acting as physical barrier to colonizing microbes but it is also a central component of host defenses through its own antimicrobial activity and its communication with underlying immune tissues [41]. The microbiota influences many of the functions of the epithelium [42]. From the prospective of barrier function development, the acquisition of the microbiota drives the production of mucus and stimulates epithelial production of certain antimicrobial peptides (AMP) [21, 43]. Within the intestine, mucus thickness reflects bacterial load, with the mucus layers in the colon being the thickest as this site has the highest bacterial burden along the gastrointestinal length [44]. Tolerance to the microbiota to limit pathological inflammation is thought to develop through a number of mechanisms. In mice, restriction of epithelial innate pattern recognition receptor activation driven by the microbiota, and in particular Toll-like receptor signaling, occurs via proteasomal degradation and microRNA-mediated inhibition of downstream signaling cascade components [45]. In human intestinal epithelial cells, reduced TLR expression [46] and negative regulation of NF-κB activity by commensal microbes have been demonstrated [47–49] and this is proposed to help mediate tolerance to the microbiota. The epithelium is therefore constantly responding to microbial cues and its function is attuned to establish symbiosis with the microbiota.

Neutrophils are essential for protection against infection and nearly every aspect of the neutrophil life-cycle is now thought to be influenced by signals from the microbiota [5, 6]. The development of the microbiota from its neonatal to adult composition stimulates the production of neutrophils in the bone marrow [15]. This occurs through microbiota-mediated stimulation of intestinal IL-17A that leads to increased levels of circulating granulocyte colony stimulating factor that promotes neutrophil production [15]. Then, upon release of these neutrophils into the circulation, the influence of the microbiota continues. In the absence of the microbiota, the ability of circulating neutrophils to enter host tissues in response to inflammation is diminished, as is the bactericidal activity of these cells [14, 50]. In addition, in the absence of cues from the microbiota, the rate of neutrophil apoptosis is accelerated [51], underlining the comprehensive influence that the microbiota has on neutrophil biology and the ability of these cells to control microbial infection.

Macrophages and dendritic cells, often collectively referred to as mononuclear phagocytes, form a critical collection of cells that are central to host defenses to infection [52, 53]. Macrophages are found in tissues throughout the body. In addition to their role in host defense, they maintain tissue homeostasis and repair damage in injured tissues. Macrophage phenotype and function is exquisitely attuned to the physiology of their host tissue. For example, in the intestine, macrophages have dampened responses to microbial stimulation that reflects the need to tolerate the enormous microbial load in the intestine [54], and in the lung alveolar, macrophages are required to prevent the accumulation of pulmonary surfactants needed for correct lung function [55]. The instructions that ensure that tissue macrophage function is correctly aligned with host tissue biology are not solely derived from the host. Within the intestine the number and function of macrophages is shaped by the microbiota. The preponderance of intestinal macrophages are derived from circulating monocytes and this process of replacement is driven by the microbiota through CCR2 activity [56]. Functionally, microbiota-derived short-chain fatty acids (SCFA) dampen the inflammatory response of intestinal macrophages while enhancing their antimicrobial activity [57, 58]. Similarly, the microbiota also primes the antimicrobial activity of intestinal macrophages through induction of cytokines such as IL-36 to control pathogen levels in the intestine [17]. Likewise, in the lung, the antimicrobial activity of alveolar macrophages is enhanced by the presence of the microbiota but the number of macrophages there, and in other extra-intestinal tissues, seems to be somewhat less influenced by the microbiota [13, 59, 60]. The function of dendritic cells in different host tissues is similarly influenced by the microbiota. Within the intestine a variety of microbiota-derived molecules have been found to influence the function of dendritic cells. Like macrophages, SCFA have been shown to reduce inflammatory cytokine production by dendritic cells thus promoting the induction of regulatory T cells (Tregs) [61]. Likewise, one of the capsular polysaccharides produced by the commensal *Bacteroides fragilis* (PSA) drives the production of the anti-inflammatory cytokine IL-10 that in turn leads to the induction of Tregs [62]. The microbiota also exerts influence on dendritic cells outside of the intestine. In the absence of the microbiota, during respiratory infection by influenza the migration of dendritic cell from the lung to the mediastinal lymph nodes is reduced, and this is associated with reduced T-cell responses and reduced anti-influenza antibody responses [63]. In addition, the microbiota regulates the activity of lung dendritic cells to make them better able to induce IgA class-switching in B cells resulting in better responses to intranasal vaccines [64]. Dendritic cells in non-mucosal tissues also have their function impacted by the microbiota. Cytokine production, and in particular interferon production, in splenic dendritic cells is enhanced by the microbiota and this promotes natural killer cell-mediated protection against systemic viral infection [59]. As these examples demonstrate, the microbiota, and in particular the intestinal microbiota, has a significant impact on the development and functional programming of innate immunity in tissues throughout the body.

The inflammatory set-point established by the microbiota is therefore important for the elimination of pathogens and prevention of infection. Equally important, however, is the regulation of immunity to prevent overt responses that can lead to loss of tolerance to commensals, inflammation, autoimmunity, or increased risk of infection and cancer [65]. Maintenance of homeostasis is therefore critical within the gastrointestinal tract where the greatest abundance of microbes engage with host immune cells. Members of the lymphocyte lineage regulate much of this control over the immune system. In generating proinflammatory responses, Th17 cells are important producers of IL-17 that activate epithelial cells to produce AMP, enhance immune responses, and recruit neutrophils to control infections [66–69]. The microbiota have also been shown to be important in the development of these T cells. Segmented filamentous bacteria have demonstrated the ability to induce Th17 accumulation by promoting IL-1β and IL-23 production from phagocytes [69, 70]. Similarly, ATP from the microbiota promotes Th17 differentiation [70]. An important counterpart to Th17 cells are Tregs that are an essential regulator of immunity through the production of anti-inflammatory mediators such as IL-10 [71, 72]. Members of the microbiota have also been shown to support the development of Tregs. Commensal Clostridia, in particular clusters IV and XIVa, enriched TGF-β and accumulation of Treg in the colon [73]. This intimate balance between an inflammatory and regulatory immune response driven by these T-cell populations is underscored by the fact that Tregs, Th17 cells, and innate lymphoid cells (ILC) 3 all share a common nuclear receptor (retinoic acid-related orphan receptor γt) and that differentiation requires a balance of signals to determine the functional fate [74]. Understanding this balance between immune responses driven by these lymphocytes is complex but is thought to play a critical role in the development of diseases such ulcerative colitis and Crohn’s disease where imbalance of immune responses to microbiota, diet, and self-antigens leads to chronic inflammation and damage. ILCs are critical cells that coordinate the activity of innate and adaptive immune cells within the gut through their production of these important signaling molecules. While the complexity of ILC functions in the gut is not fully understood, there is some evidence that the microbiota is important for regulating their functions. ILC3 limit effector T-cell responses to the microbiota, as well as being a major source of IL-22 within the intestine. Thus, their loss results in dysregulated adaptive immune responses and inflammation toward commensals [75–77]. Epithelial production of IL-25 in response to the microbiota helps dampen the inflammatory products of ILC3 and IgA production promoted by ILC2 is regulated by the microbiota [78, 79]. Sensing of microbial signatures by macrophages leads to their production of IL-1β that induces ILC3 to feedback to mononuclear phagocytes instructing IL-10 production and subsequent expansion of Tregs promoting greater immune tolerance [80]. The microbiota is therefore constantly interacting with innate and adaptive immune cells in the intestine and this controls the inflammatory tone and the set-point of intestinal antibacterial defenses. Harnessing the protective power of the intestinal microbiota therefore represents an exciting opportunity to treat infection. The intestinal microbiota is also, however, a reservoir of many pathogens that go on to cause acute infection [81]. Below, we outline the delicate balance that exists in the intestine between the beneficial microbiota and some of the most problematic antibiotic-resistant pathogens for human health.

## The intestinal microbiota and antibiotic-resistant pathogens

For many bacterial pathogens, including *Klebsiella pneumoniae*, vancomycin-resistant enterococci (VRE), and *Clostridioides difficile*, colonization of the intestinal tract is the first step on the pathway to acute infection [3, 36, 81, 82]. Despite these three pathogens ultimately causing a different spectrum of diseases, colonization of the intestinal tract is a shared point in their pathogenesis [36, 81]; thus, understanding this key step in disease could lead to treatments that are broadly effective against multiple pathogens.

*Klebsiella pneumoniae* is a Gram-negative Proteobacteria belonging to the family *Enterobacteriaceae*. It is commensal of the gastrointestinal and upper respiratory tract in several mammals, including humans, with human carriage estimated to be around 3–8% [83, 84]. In hospital patients, the incidence of gastrointestinal colonization can be significantly higher (up to 38%) [85]. Beyond its human host, it is also found in environmental reservoirs such as water, sewage, and soil [86]. In a fraction of its hosts, *K. pneumoniae* causes respiratory, urinary tract, wound, and bloodstream infections [87, 88]. Classically, it has been considered an opportunistic nosocomial pathogen with higher incidence in neonates, the elderly, immunocompromised, and notably patients receiving antibiotic treatment [84]. This view of *K. pneumoniae* as a purely nosocomial pathogen has, however, been reevaluated with a growing number of infections now also being identified in the community and not solely in patients with comorbidities [89]. *K. pneumoniae* capable of infecting these mostly healthy patients in a community setting is thought to be more virulent, and is associated with specific capsule types [90]. The development of antibiotic resistance has also amplified the importance of *K. pneumoniae* infections. Following the first European case of an extended-spectrum β-lactamase in 1983, carbapenems became vital in treating these resistant organisms [91]. However, resistance to these last-line defense antibiotics has expanded where carbapenem-resistant *K. pneumoniae* (CR-Kp) clinical isolates rose from 1.6% in 2001 to 10.4% in 2011 [91]. Similar rates of CR-Kp have increased in China from 2.4 to 13.4% between 2005 and 2016. The Centers for Disease Control and Prevention have thus declared the emergence of these carbapenem-resistant *Enterobacteriaceae* an urgent threat to public health owing to the lack of treatment options and high fatality rate. Gastrointestinal colonization is the pivotal step in *K. pneumoniae* pathogenesis [85]. Studies examining *K. pneumoniae* carriage and subsequent infection have attempted to identify factors that predispose a patient to colonization [92]. Duration of hospital exposure and usage of antibiotics increase the rate of *K. pneumoniae* colonization [92]. Length of stay is likely to increase the probability of a patient being exposed to the bacteria via healthcare workers and fomites. Antibiotic usage causes dysbiosis of the microbiota providing favorable conditions for *K. pneumoniae* [17, 93, 94]. Limited data are available on which antibiotics predispose to *K. pneumoniae* colonization but some clinical studies have demonstrated a correlation between vancomycin treatment and subsequent *K. pneumoniae* colonization [93, 94]. Supporting this idea vancomycin addition to a simulated intestinal microbial system allowed the expansion of *K. pneumoniae* [95]. The utility of the microbiota for preventing *K. pneumoniae* colonization is supported by studies where fecal microbiota transplant has been shown to promote the clearance of *K. pneumoniae* from the intestine in both humans and animal models [17, 96, 97]. Studies using animal models have shown that of the major bacterial phyla in the intestinal microbiota, it is members of the Bacteroidetes, but not the Firmicutes, Proteobacteria, or Actinobacteria, that protect against *K. pneumoniae* colonization [17]. Bacteroidetes provide protection via the stimulation of the intestinal immune system. Specifically, the development of Bacteroidetes post weaning primes the production of intestinal IL-36γ that stimulates a macrophage-dependent barrier against *K. pneumoniae* [17]. This priming of intestinal IL-36 requires that Bacteroidetes intimately associate with the intestinal mucosal and this is dependent on the Bacteroidetes commensal colonization factors that promote the association of these organisms with the mucosa [17]. Other studies demonstrate that protection against *K. pneumoniae* by the microbiota is not limited to indirect stimulation of the immune system but can also occur via direct inhibition from the microbiota. Specifically, production of SCFA by the microbiota leads to intracellular acidification of *K. pneumoniae* thus inhibiting its growth [98]. Within the other major human niche of *K. pneumoniae*, the upper airway, the microbiota is seemingly less able to prevent colonization. Again, in animal models, after direct inoculation of *K. pneumoniae* into the upper airway *K. pneumoniae* is able to successfully compete with the upper airway microbiota and establish colonization [17]. Successful colonization of the upper airway requires production of the capsular polysaccharide [17]. Capsular polysaccharide is a well-established virulence factor important for evading innate immune defenses during acute infection [88]. There is a multitude of different capsule types produced by different strains of *K. pneumoniae*. Capsular composition has a designated K nomenclature based on the K-antigen, of which there has been thought to be a total of 77 different types [99]. Originally done using agglutination, the advancement of technologies for whole genome sequencing suggests there may be even more than 138 distinct K types [100]. Importantly, the vast majority of the infections in the community are from hypervirulent strains of the serotype K1 and K2 capsule types only [101–103]. The capsule may support initiating gastrointestinal colonization but is seemingly unimportant for persistent intestinal colonization, unlike its established role in colonization in the urinary tract or its contribution to immune evasion [104–108]. In the absence of the capsule, *K. pneumoniae* can no longer compete with the upper airway microbiota and is cleared from this site [17]. Clearance is driven by the stimulation of innate immunity by commensal Proteobacteria in the upper airway, rather than Bacteroidetes, and via IL-17A activity, rather than IL-36 [17]. If *K. pneumoniae* does manage to evade the intestinal microbiota and immune defenses that inhibit its colonization, it can go on to cause lung infection. Nevertheless, the intestinal microbiota is still important in resisting disease in this distal tissue. Members of the intestinal, and upper airway, microbiota that are potent stimulators of the pattern recognition receptor Nod2 enhance early innate clearance of *K. pneumoniae* from the lung by alveolar macrophages through a IL-17A, GM-CSF, and ERK-dependent pathway [13]. This highlights the complexity of the interaction between the microbiota, immune system, and *K. pneumoniae*. At different mucosal sites there are different commensal groups that are having different effects on the immune system that may, or may not, lead to resistance to colonization and thus infection. Enterococci are part of the human microbiota but two members of this genus are significant human pathogens: *E. faecalis* and *E. faecium* [109–111]. Enterococci are the major Gram-positive coccus in the human intestinal microbiota and commensal strains can be present at levels of up to 10^7^ CFU/g in feces [110, 111]. These organisms are naturally resistant to many antibiotics and can easily acquire further resistant elements leading to many strains being multidrug resistant. Disease-causing enterococci are responsible for systemic infections, urinary tract infections, and endocarditis [109, 112]. A feature of enterococcal biology is that strains associated with disease are often markedly different to commensal enterococci. Commensal strains have smaller genomes and disease-causing strains having a broader repertoire of carbohydrate utilization pathways allowing use of host-derived glycans [110, 113, 114]. This may underlie why commensal strains rarely cause disease. It has been known for a number of years that antibiotic therapy facilitates the expansion of VRE in the intestine [115, 116]. Of particular note is that human and animal studies have found that the suppression of VRE is particularly associated with anaerobic bacteria indicating that these commensals are particularly important in keeping the levels of enterococci in check [116, 117]. There have been a number of pathways delineated by which the microbiota provides protection against VRE expansion. Protection has been shown to be via both the stimulation of the immune system and also through the direct antagonism of commensals against VRE. Microbiota-mediated resistance to intestinal VRE colonization driven by the immune system can occur via the stimulation of the innate immune system. During homeostasis the microbiota provides basal stimulation to the innate immune system through agonism of pattern recognition receptors, fortifying intestinal antimicrobial defenses [43]. In the intestine, the microbiota activates Toll-like receptors on stromal cells that stimulate the production of the AMP REG3γ [43], indicating that while the innate immune system in the intestine has its activity restrained to prevent pathological inflammation its responsiveness is not eliminated. Simple restoration of a pure TLR agonist was sufficient to stimulate this immune defense pathway and protect against VRE [43]. There are further more indirect immunological defenses that restrict enterococcal expansion through the activity of the IL-22 receptor. Signaling through the IL-22 receptor has been proposed to limit the levels of intestinal enterococci by promoting the fucosylation of host glycans [118]. It is thought that these act as a nutrient source for intestinal anaerobes, such as Bacteroides, promoting their expansion thereby limiting enterococcal numbers [118]. Further studies have begun to delineate at lower taxonomic levels specific members of the microbiota that protect against VRE. By comparing mouse microbiota that provide different levels of protection against VRE colonization, and then isolating commensals from the protective microbiota by culturing, a simple consortium of four commensals was identified that protect against VRE [11]. This consortium consisted of *Clostridium bolteae*, *Blautia producta*, *Bacteroides sartorii*, and *Parabacteroides distasonis*. Within this consortium it is *Blautia producta* that is directly antagonistic to VRE through the elaboration of a lantibiotic that is inhibitory to VRE [119]. Similarly, in patients, the enrichment of lantibiotic genes was associated with reduced *E. faecium* being detected in their stool [119]. Enterococci are also thought to be directly antagonistic to one another through the production of bacteriocins. *E. faecalis* strains that produce bacteriocins from the pPD1 plasmid are able to outcompete non-bacteriocin producing strains in the intestine, including VRE [120].

Of all infections associated with the disruption of the microbiota, it is *Clostridioides difficile* infection that is perhaps the most well known and clinically the most common [121, 122]. *C. difficile* is an obligate, Gram-positive anaerobe that exists in two states: a vegetative state, which is the metabolically active form of *C. difficile*; and the spore form, which is the dormant state used by *C. difficile* to survive harsh environmental conditions often during host-to-host transmission [123]. *C. difficile* causes a range of diseases from asymptomatic intestinal colonization through to fulminant colitis [122, 123]. Again, like the other pathogens discussed in this review, animal models and human studies have demonstrated that both the microbiota and immune system are important in controlling *C. difficile* infection. While most antibiotics that disrupt the microbiota can increase susceptibility to *C. difficile*, it is thought that fluoroquinolones, clindamycin, and cephalosporins are the major antibiotics that render patients susceptible to *C. difficile* [124, 125]. Diet composition has also been shown to be important in microbiota-mediated protection against *C. difficile* infections. Excess zinc has been shown to increase susceptibility and severity of *C. difficile* infections by altering the microbiota and reducing the effectiveness of calprotectin reduces zinc availability to the pathogen [126]. Increasing dietary carbohydrates has been shown to affect the microbiota leading to greater SCFA production that is inhibitory to *C. difficile* [127, 128]. A diet rich in carbohydrates instead of protein selects for organisms in the microbiota, such as the *Lachnospiraceae*, that are able to metabolize these carbohydrates but also the rarer amino acids that limit the availability of amino acids that *C. difficile* requires [129, 130]. A case study has supported how dietary intervention with a specific carbohydrate prevented *C. difficile* reinfection in patients [131]. *C. difficile* has two major virulence factors that are critical determinants of acute disease; these are toxin A and toxin B [132]. These proteins can inactivate rho GTPases that in intestinal epithelial cells causes cell death and thus ultimately barrier disruption [132]. Signaling through Toll-like receptor and Nod-like receptors, Myd88, and the cytokines IL-22 and IL-17A has been shown to be required for immune protection against *C. difficile* [123, 133–136]. In addition to innate immunity in the stromal cells of the intestine, resistance to *C. difficile* requires ILC, specifically ILC1, and multiple granulocytes (neutrophils and eosinophils) [137, 138]. One of the major risk factors for *C. difficile* infection is prior antibiotic therapy [122]. Initial treatment of infection often relies on the antibiotics metronidazole and vancomycin [122, 139]; however, recurrence of infection can occur in up to 35% of patients and has been associated with immunosuppression and further antibiotic therapy [140, 141]. While still not generally used as an initial treatment for *C. difficile*, fecal microbiota transplantation is used to treat recurrent *C. difficile* infection and it is perhaps the best practical demonstration of the power and utility of microbiota-based therapeutics to treat human disease, with success rates of up to 90% [142, 143]. There are, however, a number of potential drawbacks including related to the uncharacterized nature of the fecal material used for transplantation that could result in the transfer of potential pathogens or the implantation of a microbiota that has deleterious effects on host metabolism [144]. Because of the limitations of using fecal material there have been efforts to understand whether there are specific members of the microbiota that are the key protective commensals preventing *C. difficile* infection, and also to understand the mechanisms by which the microbiota prevents *C. difficile* infection that could be a more refined way to protect against *C. difficile*. Mechanistically, there have been a number of stages in the process of *C. difficile* pathogenesis that the microbiota has been shown to be inhibitory. First is through commensals out competing *C. difficile* for nutrients. After antibiotic treatment, depletion of commensals that normally consume host-derived sialic acids and succinate limiting their availability leads to an outgrowth of *C. difficile* because of the ready availability of these molecules as a nutrient source [145]. Expression of genes involved in host *N*-linked glycosylation has been demonstrated to be regulated by IL-22. Such glycosylation supports the growth of *Phascolarctobacterium* that are efficient at utilizing these host molecules and luminal succinate thereby diminishing their nutritional availability to *C. difficile* [146]. Second, the microbiota protects against *C. difficile* through the modification of another set of host-derived molecules, bile acids. Bile acids are a family of molecules that facilitate fat digestion and absorption, and within the intestine these molecules can undergo chemical transformation by members of the microbiota [147]. Bile acids unmodified by commensal bacteria are referred to as primary bile acids, whereas those that have undergone processing by commensals are referred to as secondary bile acids [147]. The relationship between bile acids and *C. difficile* is complex. Spore germination is thought to be promoted by cholic acids, whereas chenodeoxycholic acids are inhibitory to spore germination [148]. Taurocholic acid is deconjugated by bile salt hydrolases produced by commensals and it has been demonstrated using mouse models whose intestinal conditions are permissive to *C. difficile* growth due to antibiotic treatment that can be reversed by treatment with bile salt hydrolase producing bacteria [149]. This likely reduces the levels of taurocholic acid therefore reducing the germination signal for *C. difficile* limiting its expansion. Another study has shown that modification of primary bile acids by the activity of dehydroxylating enzymes also prevents *C. difficile* infection by inhibiting growth [150]. This work identified a key member of the microbiota important for this dehydroxylation step, *Clostridium scindens*, which was associated with protection against *C. difficile* in humans and was effective at preventing *C. difficile* infection in mice [150]. Similarly, other murine studies have found that protection against *C. difficile* does not require a complete, diverse microbiota and that a simple consortium of six commensals could protect against *C. difficile* infection [151]. This limited commensal consortia approach is supported by a Danish study in humans where patients were given a consortium of 10–12 commensals spanning the major human intestinal microbiota phyla (including three members of the Bacteroidetes, two members of the Proteobacteria, and seven members of the Firmicutes) that were shown to be effective at curing *C. difficile* in approximately 64% of patients [152]. This, again, highlights the utility of the microbiota in protecting against *C. difficile* and establishes the feasibility of simple, defined commensal consortia to protect against disease. The mechanistic basis by which this simple consortium is protective in humans is currently unknown. If this were deduced it could allow redesign of the consortium to attempt to increase the cure rate.

## Conclusion

**Fig. 1:**
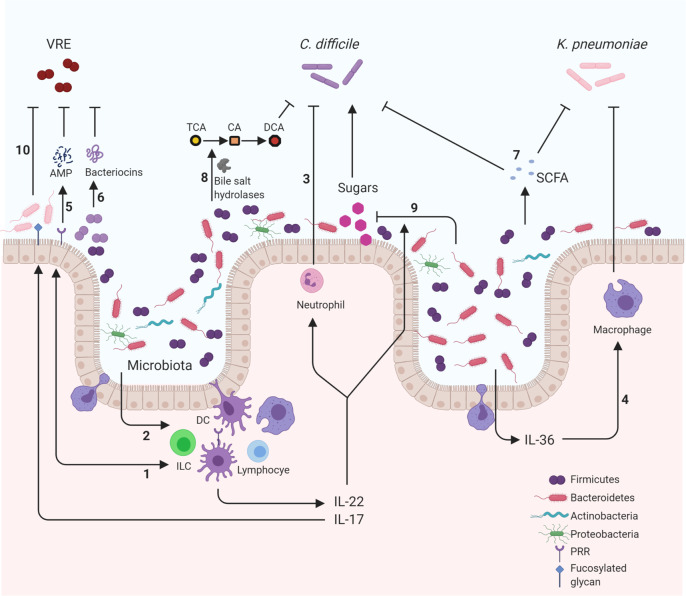
Mechanisms of microbiota-mediated colonization resistance against pathogens within the gastrointestinal tract. Representation of pathways involving components of the immune system and microbiota that help suppress gastrointestinal tract colonization by *Clostridioides difficile, Klebsiella pneumoniae*, and vancomycin-resistant enterococci (VRE). Suppressive mechanisms involve a variety of important pathways that can be broadly classified into four interconnected categories. Cellular interaction: microbiota activating intestinal epithelia (1) or immune cells (2) induce production of proinflammatory cytokines such as IL-22 and IL-17. These cytokines promote clearance of *C. difficile* through the regulation of important innate cells such as neutrophils (3). Important phyla such as Bacteroidetes induce the production of IL-36 that promotes macrophage-mediated clearing of *K. pneumoniae* (4). Antimicrobial production: pattern recognition receptor (PRR) detection of the microbiota induces the production of antimicrobial peptides (AMP) such as REGIIIγ that is protective against VRE (5). Members of the microbiota can also produce their own AMPS such as bacteriocins. For example, *Blautia producta* produce lantibiotics that inhibit VRE (6). Metabolic: metabolic products from the microbiota, including short-chain fatty acids (SCFA), can be antagonistic to pathogens reducing the fitness of *C. difficile* and acidifying *K. pneumoniae* intracellularly (7). Enzymes produced by the microbiota can also metabolize host compounds into products that are disruptive to pathogens. The bile acid taurocholic acid (TCA) is deconjugated by microbiota bile acid hydrolases into cholic acid (CA) and subsequently into deoxycholic acid (DCA) that is inhibitory to *C. difficile* growth (8). Nutritional immunity: nutrients are limited resources and so utilization by the microbiota diminishes availability to incoming pathogens. IL-22-induced *N*-glycosylation promotes microbiota that utilize sialic acid and succinate reducing their abundance preventing the expansion of *C. difficile* (9). Similarly, IL-22 induction of glycan fucosylation promotes anaerobic commensals competing with VRE limiting its expansion (10). These examples demonstrate how the microbiota provide resistance to three pathogens by engaging with a multitude of mechanisms that are antagonistic to the success of the pathogens in the gastrointestinal tract. DC dendritic cell, ILC innate lymphoid cell. Created with BioRender.com.

The examples outlined above demonstrate that the microbiota protects against numerous major human bacterial pathogens at multiple stages in their pathogenesis (Fig. 1). The microbiota is therefore an important and highly effective component of host defenses against infection. It is, however, a component of host defenses that is exquisitely sensitive to disruption. A common thread throughout the work described above is that even a transient disruption to the microbiota can eliminate some of its protective effects. This is perhaps most evident during, and even post, antibiotic treatment that can lead to pathogen colonization and expansion, especially in the intestine. The presence of the microbiota makes colonization almost impossible for many bacterial pathogens but these same pathogens can reach levels many orders of magnitude greater after only a single dose of antibiotics that disrupt the microbiota. This bacterial bloom in the gut can have devastating consequences for the host, as data now support that it is often the expansion of these colonizing pathogens that proceeds more serious acute infections [85, 153]. Understanding how bacterial pathogens can so rapidly capitalize of these defects in microbiota-mediated defenses may therefore provide unexplored avenues of investigation to develop new ways to prevent infections by these organisms. Despite their obvious importance and utility, another consistent theme emerging from many studies of these pathogen is that antibiotic overuse can cause multiple problems for patients. First is the well-characterized selection for resistant strains of *K. pneumoniae*, VRE, and *C. difficile* and second is the removal of the protective barrier formed by the microbiota. This only emphasizes further need for more judicious use of antibiotics and the development of alternative approaches, such as using microbiota-based therapeutics to combat bacterial pathogens. A further theme apparent from the mechanistic studies of microbiota-mediated protection against different pathogens is that for a given pathogen the microbiota provides a number of layers of host defense. For example, for *K. pneumoniae*, the intestinal microbiota protects against intestinal colonization by *K. pneumoniae* through direct antibacterial antagonism [98] and through the enhancement of IL-36-mediated defenses [17]. Furthermore, if *K. pneumoniae* go on to cause acute lung infection, signals from the intestinal microbiota enhance respiratory innate immunity too [13]. Thus, multiple aspects of *Klebsiella* pathogenesis are controlled by the microbiota that could be important for the design of protective commensal consortia. It would be advantageous to design consortia where each member provides this multilayered protection. For example, Bacteroidetes that are also potent activators of the Nod-like receptors would provide protection against both intestinal colonization and pneumonia.

To fully utilize the microbiota to protect against infections there are a number of areas that need to be understood in greater detail. As outlined above, there are an increasing number of preclinical studies that have identified microbiota members that militate against infectious disease; however, one of the biggest hurdles that currently prevent these organisms being pursued further is the difficulties in engrafting them into a new microbiota [154]. This problem is similar to the process of antibiotic drug discovery whereby promising candidate small molecules identified to be inhibitory in vitro fail because of their poor pharmacological properties in vivo. The ability of a microbiota to resist the entry of new microbial members, colonization resistance, is therefore not limited to resistance to pathogens and seemingly extends to myriad other non-pathogenic microbes including those able to form commensal relationships with mammals. A recent study of human colonization by a number of popular probiotics found that there was only patchy colonization of the host after probiotic consumption [154]. To get around this issue for the microbiota means that the rules of colonization need to be better understood. How are these microbial communities assembled and maintained, and how can we use this information to circumvent the colonization resistance encountered by potentially protective commensals? It has been shown that the ability of probiotics to establish themselves at the intestinal mucosa inversely correlates with the levels of these species in the microbiota before probiotic administration [154]. This could suggest that there is some form of niche exclusion occurring, analogous to that of enterococci outlined above [120], that this is at least partly responsible for colonization resistance encountered by commensals. As discussed above, microbiota disruption by antibiotics is one of the main drivers of pathogen colonization and expansion. This could be used as an opportunity by providing a window for the introduction of protective species during or after the completion of antibiotics, thus exploiting microbiota disruption to implant protective commensals rather than letting this niche be filled by pathogenic organisms. A further issue is that the range of probiotics that are considered safe for human consumption is small, in comparison to the spectrum of microbial symbionts in the intestine [155]. The available repertoire of protective microbes is therefore limited and likely does not cover the entire range of taxa that may be important in protecting against pathogenic microbes. Increasing the pool of suitable organisms is therefore required, but this is not straightforward as it has to be ensured that they will not cause infections themselves, they will not spread antibiotic resistance, do not produce toxins, and there is no genetic drift during their propagation from parental stocks. This suggests that further consideration as to which is the best method to harness the protective effects of the microbiota therapeutically is necessary. Should we use live bacteria, bacterial components, or promote the growth of protective organisms through dietary modification? Each of these approaches comes with their own advantages and disadvantages. The upside of using live bacteria is that, if they successfully colonize the host, they can be self-maintaining and thus can provide longer-term protection compared with using components of these protective organisms that may only have a transient influence on the host because they are more rapidly excreted and are not self-perpetuating. As just described, live bacteria have a number of potential downsides therefore bacterial components or metabolites are an alternative to live bacteria, and include molecules like SCFA. The defined nature of these molecules means that dosing can be more precise and preparations of these molecules can be more consistent. There remains, however, only a limited number of molecules identified from the microbiota where their mode and full spectrum of activity is completely defined [156]. SCFA are some of the most well-characterized microbiota-derived molecules but they influence myriad cells and this pervasive activity needs to be carefully considered before they can be deployed to ameliorate diseases associated with microbiota disruption [157]. A nice example of a strategy utilizing a microbial product was observed in the attempt to deliver the bacteriocin thuricin CD to attenuate *C. difficile*. It was demonstrated that rectal delivery of thuricin CD induced clearance of *C. difficile* from the colon of mice [158]. Dietary modification could be used to promote the outgrowth of beneficial or protective microbiota members and circumvents the difficulties encountered by the use of whole bacteria or bacterial components. This approach, however, is highly dependent on the initial conditions of the microbiota. Given the complexity of the microbiota, even after disruption by antibiotics, it is hard to predict whether a given dietary modification will have the desired effect because the relationship between diet and microbiota composition is still poorly understood in detail [159]. For example, even if dietary modifications do reconfigure the microbiota, it is unclear how long these take and if this timing varies between people too widely it might render this approach not clinically useful. A further alternative is to bypass the microbiota completely and focus on the immune pathways the microbiota regulates. For example, cytokines like IL-22 and IL-36 are induced by the microbiota and protect against infection, targeting these pathways directly to enhance immunological defenses might be an effective and controlled way to combat infections. Much more needs to be understood about signaling molecules like IL-22 and IL-36, however, before this can be pursued further. For example, will this type of approach work in immunocompromised patients and how will the protective effects against infection be balanced against the potential overt inflammatory response these molecules can induce? These difficulties should not distract from the vast potential of the microbiota as an alternative means to combat infection by antibiotic-resistant pathogens. This potential will be most rapidly realized through increased mechanistic understanding of the interplay between the microbiota, immune system, and pathogenic microbes. As our understanding of how the microbiota inhibits bacterial infections increases so will our ability to use the microbiota to treat antibiotic-resistant infection.
